# Improvements in resting-state autonomic function precede clinical improvement in adolescent non-suicidal self-injury

**DOI:** 10.1038/s41398-026-04012-7

**Published:** 2026-04-03

**Authors:** Julian Koenig, Ines M. Mürner-Lavanchy, Nicole Hedinger, Silvano Sele, Michael Kaess

**Affiliations:** 1 University Hospital of Child and Adolescent Psychiatry and Psychotherapy, University of Bern, Bern, Switzerland; 2 Department of Child and Adolescent Psychiatry, Psychosomatics and Psychotherapy, Faculty of Medicine and University Hospital Cologne, University of Cologne, Cologne, Germany; 3 Faculty of Psychology, University of Basel, Basel, Switzerland; 4 Department of Child and Adolescent Psychiatry, Centre for Psychosocial Medicine, University of Heidelberg, Heidelberg, Germany

**Keywords:** Physiology, Psychiatric disorders

## Abstract

Adolescents engaging in non-suicidal self-injury (NSSI) have been reported to show altered autonomic nervous system (ANS) function, indexed by decreased heart rate variability (HRV) and increased heart rate (HR). Preliminary findings in adolescents with borderline personality disorder (BPD) traits suggest, that improvement in ANS function is longitudinally associated with clinical improvement and that ANS activity predicts clinical outcome. Existing studies, however, are limited by small sample sizes and short follow-ups. N = 227 help-seeking adolescents with NSSI disorder who presented to an early intervention service for BPD participated in baseline and two yearly follow-up assessments (N_FU1_ = 81, N_FU2_ = 37), including comprehensive clinical diagnostics as well as recordings of resting electrocardiography. Associations between HR, HRV and clinical outcomes of interest (i.e., NSSI frequency, depression severity, number of BPD criteria and global functioning) were examined using structural equation modelling. While multivariate models showed no evidence for an association between HRV or HR and any of the clinical outcomes, there was evidence from models including cross-lagged effects, that HRV and HR predicted depression severity, number of BPD criteria (HRV only) and global functioning at subsequent assessments. This effect was not observed for NSSI frequency. Improvements in ANS function, indexed by an increase in HRV and decrease in HR, seem to precede the improvement of clinical symptoms in adolescents engaging in NSSI. Findings have clinical implications, suggesting that targeting ANS function as adjuvant treatment in adolescents engaging in NSSI is warranted, and routine monitoring of ANS function my guide clinical decision making.

## Introduction

Non-suicidal self-injury (NSSI; i.e., the self-directed act of harming one’s own body tissue by cutting etc. without suicidal intent) is common in adolescents [1]. Research addressing neurobiological mechanisms promoting the development and maintenance of the behavior [2] suggest alterations of endogenous stress-response systems, including the autonomic nervous system (ANS). We have previously shown, that heart rate variability (HRV), a proxy of parasympathetic vagus nerve activity, is decreased, while heart rate (HR) is increased in adolescents engaging in NSSI. These changes correlate with the severity of borderline personality disorder (BPD) symptoms and the current level of functioning [3]. This pattern was confirmed in an independent study, suggesting that ANS dysfunction, indexed by decreased HRV and increased HR, is associated with symptom severity in general rather than specific symptomatology in adolescents with BPD [4]. In a preliminary longitudinal study, we were able to show that changes in resting-state HRV and BPD pathology covary over time, such that a decrease in BPD severity was associated with an increase in HRV in adolescents engaging in NSSI [5]. Further research illustrated that pre-treatment HRV significantly predicted clinical improvement (i.e., a decrease in BPD pathology and increase of global functioning) over time [6] in adolescents with BPD receiving dialectical behavioral therapy. Findings were replicated in an independent, naturalistic study outside of standardized treatment [7].

Although ANS dysfunction seems to be (1) robustly altered in adolescents engaging in NSSI, and (2) is associated with the course of symptoms over time, existing evidence is limited by small sample sizes, short follow-up time and missing adjustment of important confounding variables of ANS function, including body mass index (BMI) and health-related behavior (i.e., smoking, alcohol intake). Although the temporal dynamics linking autonomic regulation and clinical trajectories have not yet been conclusively clarified, emerging longitudinal findings suggest that autonomic alterations may precede subsequent changes in clinical symptomatology. Our hypothesis, that changes in HRV precede the improvement of clinical symptoms, was based on a review of prior longitudinal evidence, and theoretical models [8], supported by results from HRV biofeedback studies, which show that changing HRV can improve depressive symptoms in the long run [9], and studies on vagus nerve stimulation for the treatment of depression [10].

Here we aimed to replicate and extend existing findings, addressing the association of HRV, HR and clinical symptoms in a large longitudinal sample of adolescents engaging in NSSI. Based on emerging longitudinal findings [11, 12], it was hypothesized, that improvement in ANS dysfunction, indexed by a re-increase in HRV and a re-decrease in HR, would precede the improvement of clinical symptoms (i.e., depression severity, number of BPD criteria, frequency of self-injury and global functioning).

## Methods

### Participants

Data for the present analyses stem from the outpatient clinic for adolescents with risk-taking and self-injurious behavior (AtR!Sk; Ambulanz für Risikoverhalten und Selbstschädigung) at the Department of Child and Adolescent Psychiatry of the University of Heidelberg [13]. Clinical data were acquired in a larger AtR!Sk-Cohort study, whereas neurobiological data has been derived from the add-on AtR!Sk-Bio study. Study protocols of the AtR!Sk-Cohort study (ID: S-449/2013) as well as of the AtR!Sk-Bio study (ID: S-514/2015) were approved by the local ethics committee of the Medical Faculty at the University of Heidelberg. Both investigations were conducted in accordance with the declaration of Helsinki [14]. Patients were excluded on the basis of an insufficient understanding of German language. In addition, patients with acute psychotic symptoms, acute suicidality, severe cardiovascular diseases and autoimmune diseases had to be excluded. All patients and their legal guardians provided written informed consent prior to inclusion in each study.

All patients included in the present sample had undergone an initial routine diagnostic baseline assessment (T0), carried out by experienced clinical professionals within the course of regular clinical care at the AtR!Sk outpatient clinic. Following the T0 assessment patients received individual treatment offers within a stepped-care framework based on the severity of BPD pathology [15]. Clinical assessments were repeated on an annual basis to track long-term development and clinical outcome. In 2016, the add-on AtR!Sk-Bio study was implemented, and patients were subsequently recruited following T0 for additional assessments. For their participation in the AtR!Sk-Bio assessments, patients received an allowance of 40€.

### Procedure and measures

Baseline and annual follow-up assessments (+/- two months) took place at the Clinic for Child- and Adolescent Psychiatry, University Hospital Heidelberg. For the present study, data at baseline as well as one- and two-year follow-ups were analyzed. At each time point, patients underwent two separate appointments.

The first appointment consisted of a sociodemographic anamnesis and several clinical diagnostic instruments. Relevant instruments for the present study are the *Self-Injurious Thoughts and Behaviors Interview* SITBI-G [16] to assess the number of days on which patients engaged in NSSI, the *Structured Clinical Interview for DSM-IV-Axis* SCID-II [17] for the assessment of the number of BPD criteria fulfilled, and the *Depression Inventory for Children and Adolescents* (DIKJ) [18] for dimensional depressive symptomatology. Baseline and annual follow-up assessments (+/- two months) took place at the Clinic for Child- and Adolescent Psychiatry, University Hospital Heidelberg between 2016 and 2019. Following diagnostic interviews, clinicians rated the *Children’s Global Assessment Scale* (C-GAS) [19].

The second appointment took place maximal six weeks after the first one and consisted of an anamnesis and several neurobiogenetic measurements, of which the measurement of cardiac function is relevant for the present study. HR was recorded at 1024 Hz during a baseline vanilla assessment consisting of a minimally demanding Color Detection Task [20] with an EcgMove 3 sensor (Movisens GmbH; Karlsruhe, Germany), attached to a chest belt with dry electrodes. Segmentation of the baseline-task-interval was performed using timestamps at the initiation as well as at the termination of the CDT as a reference. All HRV recordings were conducted in the morning (between 8:00 and 12:00) under seated resting conditions following a brief acclimatization period, minimizing variability due to diurnal rhythm and physical exertion. In line with established methodological guidelines [21, 22], acute influences such as caffeine, nicotine, or moderate physical activity were controlled through standardized morning assessments under resting conditions, minimizing short-term variability. Smoking (days with smoking during the past month) and alcohol intake (days with alcohol consumption during the past month) were assessed at baseline and included as covariates together with age, sex, and BMI. To maintain model parsimony and stability, covariate inclusion was restricted to baseline measures, as modeling time-varying covariates would have required estimating additional trajectories and their interactions, which was not feasible given the small follow-up sample size. Raw electrocardiogram (ECG) data were first screened using the UnisensViewer (Movisens GmbH; Karlsruhe, Germany). Raw data were processed using the Kubios HRV Premium software (Version 3.0) [23]. R peaks were manually corrected, accounting for movement artifacts and potential extra systoles. HR in beats per minute and the root mean square of successive differences (rMSSD) of normal to-normal intervals, as a measure of HRV, in milliseconds were derived. Data analysis was performed using the open source R package Heart Rate Variability R-HRV [24].

### Statistical analyses

The variables NSSI in the past month and HRV were log transformed (log+1; one was added to the NSSI values because zero was possible) to reduce positive skewness and approximate a normal distribution, as recommended for parametric modeling of right-skewed physiological and behavioral variables [21, 22]. Dropout analyses were conducted to examine whether baseline autonomic functioning or clinical severity were associated with attrition. Univariate latent growth curve models were used to estimate the change trajectories. We estimated the loading of the third measurement occasion to account for non-linear change patterns. Although only few patients took part in follow-up 2, both follow-up time-points were included, to allow for modelling the residual structure. Then, bivariate latent growth curve models estimated associations between HRV, HR and four clinical outcome measures (days with NSSI in the past month, depression severity, number of BPD symptoms and global functioning (GAF). Bivariate latent growth curve models allow for modelling associations between person-specific intercepts, slopes and within-person errors (e.g. if a person has a greater HR/HRV than expected at a time point, will this person also have a higher number of NSSI at the same time point?). However, as within-person errors were too large compared to the random slope variability, person-specific changes could not be reliably separated from within-person variability. Therefore, the random slope variance was set to zero and the analysis was restricted to assessing intercept-intercept and residual-residual associations. Additionally, we added lagged and cross-lagged effects (e.g., if a person has a greater HR/HRV than expected at a time point, will this person also have a higher number of NSSI at the next time point?) to the residual structure of the models. To test whether adding lagged and cross-lagged effects improved model fit, we used likelihood ratio tests. An additional cross-lagged model for a representative clinical outcome was estimated to support visual interpretation of the reported effects. In all models, age, sex, BMI, smoking (days of smoking during the past month) and alcohol intake (days with alcohol intake in the past month) at baseline were included as covariates on the intercepts and slopes. Interpretation of the findings was based on overall patterns and magnitude of differences rather than individual *P* values alone [25]. All statistical analyses were performed using Stata/SE (Version 18.0; Stata Corp LLC, College Station, TX, USA) and Mplus (Version 8.10; [26]).

## Results

### Sample characteristics

The final sample size for analyses comprised *N* = 227, with *N* = 186 female and *N* = 41 male patients. At baseline, the most frequently reported methods of NSSI were cutting (85.3%), skin scratching (29.3%), hitting oneself (25.8%), and manipulating wounds (23.6%). Sociodemographic and clinical characteristics of the sample by time of assessment are provided in Table 1. Standardized and unstandardized estimates for log-RMSSD (lag-0 and lag-1 effects) are reported in Table 2, showing that BMI, smoking, and alcohol use did not predict autonomic functioning at the same or subsequent time points. Dropout analyses indicated that dropout was not systematically associated with autonomic function or severity of symptoms.

**Table 1:** Socio demographic and clinical sample characteristics at T_0_.

	Baseline		Follow-up 1		Follow-up 2	
	M (SD) or N	Range or %	M (SD) or N	Range or %	M (SD) or N	Range or %
Demographics						
Sex (female)	186	81.9				
Age (years)	15.0 (1.44)	12.0, 17.0	16.1 (1.42)	13.0, 19.0	16.8 (1.42)	13.0, 19.0
BMI	21.2 (3.73)	13.9, 35.0	-	-	-	-
Smoking	3.08 (2.62)	1.00, 7.00	3.46 (2.62)	1.00, 7.00	3.76 (2.82)	1.00, 7.0
Alcohol intake	1.88 (1.27)	1.00, 7.00	1.95 (1.13)	1.00, 6.00	2.19 (1.29)	1.00, 6.00
Clinical measures						
NSSI past month	5.58 (7.70)	0, 30.0	1.94 (4.94)	0, 30.0	0.94 (2.73)	0, 12.0
Depression severity	27.9 (9.88)	6.00, 47.00	21.5 (11.8)	2.00, 45.00	19.6 (9.36)	4.00, 38.00
Number BPD criteria	3.06 (2.17)	0, 9	2.73 (2.26)	0, 7	2.05 (2.33)	0, 8
Global functioning	51.8 (10.0)	30.0, 90.0	66.8 (16.3)	35, 100	69.3 (17.8)	30.0, 100
Inpatient treatment (days)	-	-	13.9 (27.7)	0, 120	4.46 (18.8)	0, 90
Outpatient treatment (days)	-	-	14.2 (14.5)	0, 80	15.1 (24.4)	0, 105
Cardiac measures						
HRV	58.5 (31.6)	5.4, 189	39.5 (20.6)	5.4, 111	46.4 (25.6)	12.9, 119
HR	25.1 (11.1)	43.7, 107	80.2 (9.16)	61.1, 104	77.7 (10.1)	53.4, 94.9

Smoking = days with smoking during the last month. Alcohol = days with alcohol intake during the last month. Childhood trauma was only assessed at baseline.

**Table 2:** Standardized and unstandardized estimates for Log RMSSD (lag-0 and lag-1 effects).

Unstandardized estimates for Log RMSSD (lag-0 and lag-1 effects)				
Log RMSSD on	Estimate (B)	SE(B)	Estimate/SE (Z)	p-value
Lag-0 effects				
BMI-lag0	–0.030	0.048	–0.624	0.533
NIK3-lag0	–0.035	0.043	–0.807	0.420
ALK4-lag0	0.056	0.067	0.829	0.407
Lag-1 effects				
BMI-lag1	–0.050	0.048	–1.055	0.291
LOGRMSSD-lag1	–0.227	0.191	–1.192	0.233
NIK3-lag1	0.039	0.038	1.028	0.304
ALK4-lag1	–0.013	0.065	–0.193	0.847
Standardized estimates for Log RMSSD (lag-0 and lag-1 effects)				
Lag-0 effects				
BMI-lag0	–0.187	0.287	–0.652	0.514
NIK3-lag0	–0.233	0.278	–0.837	0.403
ALK4-lag0	0.181	0.266	0.802	0.422
Lag-1 effects				
BMI-lag1	–0.236	0.269	–0.877	0.318
LOGRMSSD-lag1	–0.226	0.188	–1.205	0.228
NIK3-lag1	0.213	0.234	0.911	0.362
ALK4-lag1	–0.043	0.220	–0.194	0.846

The first three rows represent contemporaneous effects (lag0), and the last three rows represent lagged effects (lag1).

*BMI*, body mass index; NIK3, days with smoking during the last month; *ALK4*, days with alcohol use during the last month; *Log RMSSD*, log-transformed root mean square of successive differences; *Lag-0*, effect within the same assessment wave; *Lag-1*, effect predicting the next assessment wave.

**Fig. 1:**
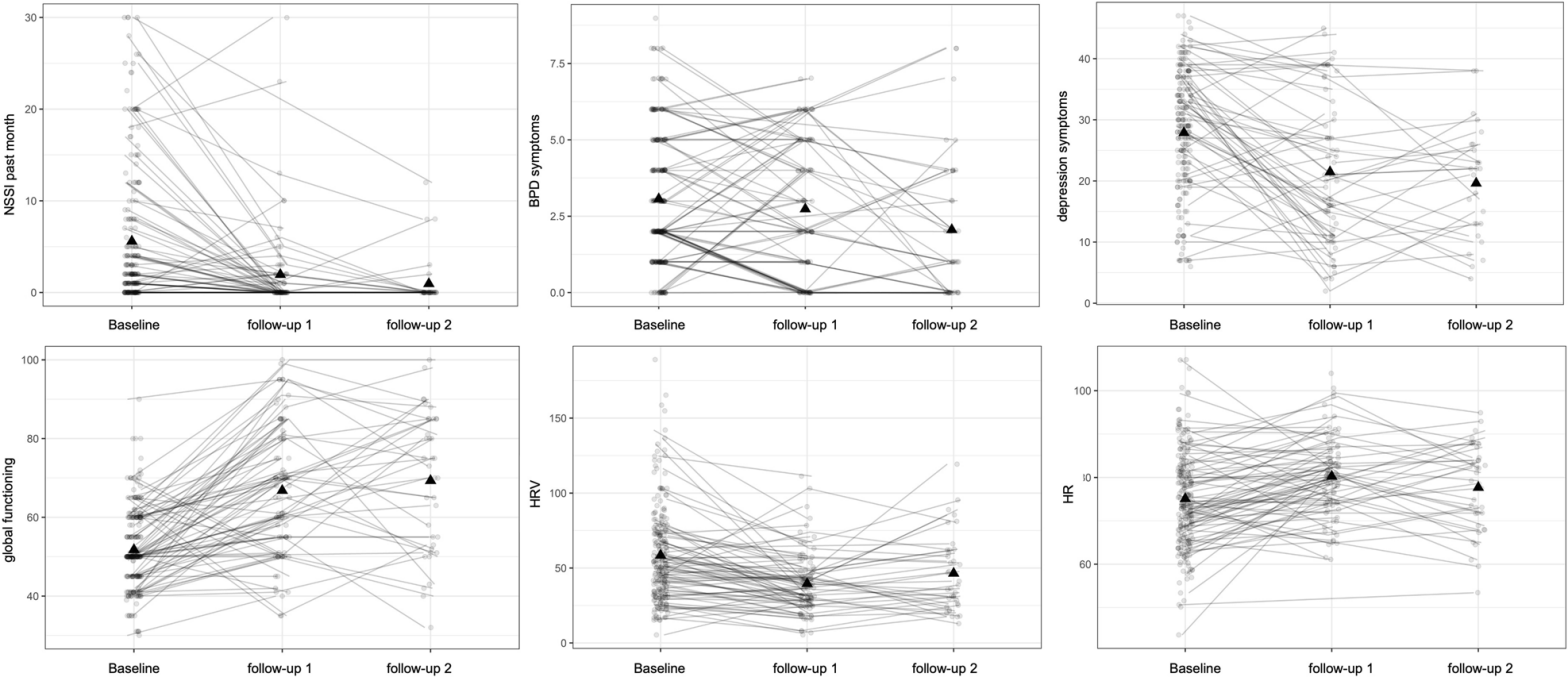
Trajectory plots for heart rate, heart rate variability and clinical variables. Individual data is shown (lines and circles), triangle represent average values per time point.

Average and individual trajectory plots of clinical variables as well as HR/HRV are depicted in Fig. 1 and results are reported in Table 3. Univariate models showed that on average, NSSI incidents in the past month, depression severity, and the number of BPD criteria decreased, while global functioning increased across time points. Average change trajectories showed a higher change from baseline to follow-up 1 than from follow-up 1 to follow-up 2. Model results further indicated that on average, HRV decreased, while HR did not change significantly across time-points.

**Table 3:** Univariate models - average change in measures.

	Intercept		Slope			Loading on FU2			Model fit		
	est	SE	est	SE	*p*	est	SE	*p*	χ^2^ p	CFI	RMSEA
NSSI past month	1.430	0.106	−0.840	0.141	*<0.001*	1.183	0.159	*<0.001*	0.658	1.000	0.000
Depression severity	29.459	0.962	−7.869	2.027	*<0.001*	1.188	0.279	*<0.001*	0.332	0.969	0.027
Number BPD criteria	2.664	0.190	−0.286	0.217	*0.189*	1.780	0.795	*0.025*	0.177	0.961	0.044
Global functioning	52.585	0.937	17.535	2.341	*<0.001*	1.198	0.207	*<0.001*	0.464	1.000	0.000
HR	77.791	1.015	1.413	1.384	*0.307*	0.285	0.282	0.312	0.742	1.000	0.000
HRV	3.825	0.053	−0.172	0.072	*0.017*	0.499	0.234	*0.033*	0.828	1.000	0.000

Unstandardized coefficients.

Additionally, the intercept and slope included several covariates: age, sex (reference level: female), BMI, smoking, alcohol intake, at baseline.

*Est* estimate, *SE* standard error, *χ*^*2*^ *p* Chi Square test of model fit, *CFI* Comparative Fit Index, *RMSEA* Root Mean Square Error of Approximation.

### Longitudinal associations between HRV and clinical outcomes

Multivariate models showed no evidence for an association between HR or HRV and any of the clinical outcomes of interest (Table 4).

**Table 4:** Random intercepts and residual associations.

	Int-Int			Res-Res-Baseline			Res-Res-Follow-up2/3			Model fit		
	est	SE	*p*	est	SE	*p*	est	SE	*p*	χ^2^ p	CFI	RMSEA
NSSI past month	−0.123	0.174	*0.479*	−0.025	0.128	*0.842*	−0.056	0.142	*0.692*	0.915	1.000	0.000
Depression severity	−0.304	0.144	*0.035*	0.099	0.159	*0.532*	0.051	0.160	*0.752*	0.255	0.961	0.028
Number BPD criteria	−0.266	0.170	*0.117*	0.287	0.136	*0.035*	0.108	0.128	*0.398*	0.048	0.924	0.050
Global functioning	0.285	0.220	*0.195*	−0.169	0.139	*0.223*	0.046	0.123	*0.709*	0.001	0.783	0.074

Standardized coefficients (correlations).

*Int*, Intercept, *Int-Int*, Intercept-intercept correlation, *Res-Res-Baseline*, residual-residual correlation at baseline, *Res-Res-Follow-up2/3*, residual-residual correlation at follow-up 2 and follow-up 3, *est*, estimate, *SE*, standard error, *χ*^*2*^ *p*, Chi Square test of model fit, *CFI*, Comparative Fit Index, *RMSEA*, Root Mean Square Error of Approximation.

**Fig. 2:**
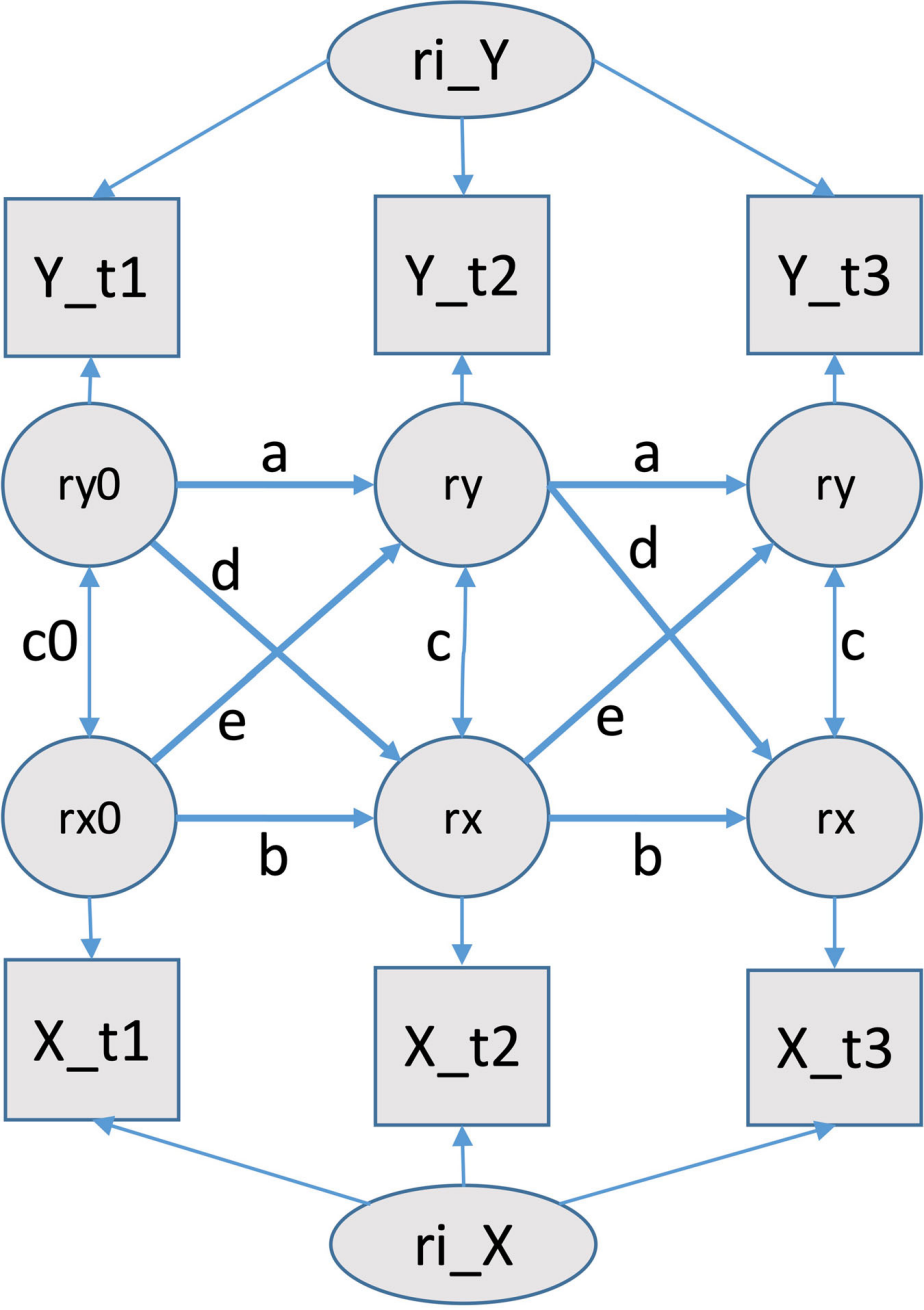
Illustration of the random-intercept cross-lagged panel model for two exemplary variables (e.g., Y = RMSSD and X = GAF). Lagged and cross-lagged effects were constrained to be equal across time. Residual variances and their covariance at follow-up time points were constrained to equality, while residuals and their covariance at baseline were freely estimated. Intercepts and covariates on the intercepts are not shown.

Models including lagged and cross-lagged effects showed no evidence of a cross-lagged association between HR/HRV and NSSI in the past month. There was evidence, however, that HRV at one time point predicted depression severity, the number of BPD symptoms, and global functioning at the next time point. Detailed model results are reported in Table 5. Corresponding effects for HR, were only observed for depression severity and global functioning, as depicted in Table 6. An illustration of the cross-lagged effects for a representative outcome is depicted in Fig. 2. The random-intercept cross-lagged panel model visualizes the within-person autoregressive and cross-lagged associations between RMSSD and GAF over time.

**Table 5:** Lagged and cross-lagged effects of heart rate variability.

	Out-on-Out			HRV-on-HRV			HRV-on-Out			Out-on-HRV			Fit		
	est	SE	*p*	est	SE	*p*	est	SE	*p*	est	SE	*p*	χ^2^ p	CFI	RMSEA
NSSI past month	0.278	0.302	*0.357*	−0.032	0.230	*0.891*	0.129	0.226	*0.567*	−0.025	0.194	*0.896*	0.872	1.000	0.000
Depression severity	0.263	0.212	*0.215*	0.046	0.230	*0.840*	−0.042	0.190	*0.823*	−0.463	0.169	*0.006*	0.523	1.000	0.000
Number BPD criteria	0.438	0.275	*0.112*	0.054	0.239	*0.821*	−0.087	0.246	*0.724*	−0.307	0.136	*0.024*	0.095	0.949	0.045
Global functioning	0.198	0.162	*0.223*	0.025	0.188	*0.893*	−0.153	0.126	*0.226*	0.462	0.123	*0.000*	0.227	0.967	0.032

Standardized regression coefficients.

*Out-on-Out*, Outcome on outcome, *est*., estimate, *SE*, standard error, *χ*^*2*^ *p*, Chi Square test of model fit, *CFI*, Comparative Fit Index, *RMSEA*, Root Mean Square Error of Approximation.

**Table 6:** Lagged and cross-lagged effects of heart rate.

	Out-on-Out			HR-on-HR			HR-on-Out			Out-on-HR			Fit		
	est	SE	*p*	est	SE	*p*	est	SE	*p*	est	SE	*p*	χ^2^ p	CFI	RMSEA
NSSI past month	0.342	0.358	*0.364*	0.157	0.253	*0.534*	−0.111	0.274	*0.686*	0.071	0.223	*0.750*	0.659	1.000	0.000
Depression severity	0.267	0.215	*0.214*	0.180	0.200	*0.368*	0.127	0.205	*0.536*	0.480	0.149	*0.001*	0.704	1.000	0.000
Number BPD criteria	0.366	0.263	*0.164*	0.105	0.235	*0.655*	−0.064	0.275	*0.816*	0.159	0.158	*0.316*	0.101	0.948	0.044
Global functioning	0.311	0.183	*0.090*	0.062	0.208	*0.767*	0.157	0.174	*0.367*	−0.288	0.137	*0.035*	0.206	0.959	0.034

Standardized regression coefficients.

*Out-on-Out*, Outcome on outcome, *est*., estimate, *SE*, standard error, *χ*^*2*^ *p*, Chi Square test of model fit, *HR-on-Out*, outcome predicting HR, *Out-on-HR*, HR predicting outcome, *CFI*, Comparative Fit Index, *RMSEA*, Root Mean Square Error of Approximation.

## Discussion

The present study sought to extend on existing evidence, addressing the association of changes in ANS function and clinical outcome in adolescents engaging in NSSI over time. Patients showed significant clinical improvement over time. Based on the group average, HRV decreased and HR showed no significant changes over the course of the study. We found evidence for cross-lagged effects, suggesting that changes in HRV – not HR – are associated with subsequent changes in depression severity, the number of BPD symptoms, and global functioning. Increases in HRV predicted a decrease in symptom severity at the following assessment. The observed temporal pattern of increases in HRV preceding subsequent clinical improvements is consistent with the theoretical assumption that vagal activity, may exert bottom-up influences on CNS processes and subsequent symptom trajectories [8]. Empirically, the present findings are in line with longitudinal findings indicating that reduced baseline HRV predicts later increases in depressive symptoms, whereas depressive symptoms do not predict subsequent HRV [11, 27]. A similar temporal pattern has been reported in treatment studies, in which HRV predicts treatment outcome in anxiety disorders [28] and depression [29, 30–34]. These findings are further supported by intervention studies showing that modifying HRV through biofeedback [9] or vagus nerve stimulation [10] may improve depressive symptoms. These findings have several implications.

First, our analyses show that the effect is specific to HRV. While HR represents mixed influences from the sympathetic and parasympathetic nervous system, our index of HRV (rMSSD) is reflective of parasympathetic vagal control of the HR. Greater vagal activity has been shown to be predictive of treatment outcome of psychotherapy [35] in other disorders, including anxiety [28], and depression [36]. While most of the existing evidence stems from studies in adults, we illustrate respective effects in underage patients from a natural help-seeking sample outside of controlled trials. Interestingly, effects were only observed on the individual subject level, as group means showed no general increase in HRV over time. We have previously illustrated that pubertal development is associated with natural occurring changes in ANS function, and adolescents in the absence of the normative increase in vagal activity indexed by HRV, may show increased risk for the development of psychiatric disorders [37]. In the absence of a control group, we can only speculate that the decrease of HRV over time on the group level (in the absence of changes in HR) reflects the normative decline in HRV towards the end of pubertal development. Individuals showing a relative increase, irrespective of the trend on the group level, showed favorable development of clinical symptoms. Large-scaled population-based studies are warranted to disentangle effects of normative pubertal maturation from those associated with psychiatric illness and treatment response.

The observed effects were specific to depression, BPD, and global functioning and not associated with changes in behavior (NSSI). This finding suggests that changes in HRV are predictive of improved emotional well-being but do not map to behavioral changes to a similar degree. Consistent with transdiagnostic models distinguishing internalizing from externalizing trajectories of dysregulation [38, 39], the differential effects observed with regard to affective and behavioral indicators of dysregulation might indicate that ANS function may reflect primarily the internalizing dimension of self-regulatory functioning, rather than behavioral dimensions such as NSSI. This interpretation thus supports the assumption that improvements in vagal regulation are associated with enhanced regulation of affective processes. However, as NSSI shows as dysfunctional strategy of emotion regulation – this distinction is less clear. More likely, the absence of longitudinal associations between ANS function and NSSI frequency may partly reflect methodological and conceptual factors. Variability in NSSI decreased markedly across follow-up assessments, which may have reduced statistical power to detect temporal associations.

Our prior cross-section and longitudinal studies are well in line with this finding [4–6], suggesting that ANS dysfunction indexed general symptom severity – in particular with respect to emotion dysregulation – and are not associated with risk-behavior and NSSI, representing dysfunction strategies to cope with dysregulated emotion. Irrespective of this, findings from the present study suggest that monitoring ANS function carries clinical value in the treatment process of adolescents with NSSI. Further, if increases in HRV are a necessity to enable therapeutic changes, adjuvant treatments targeting vagal activity could be considered in a stepped-care approach. While the present findings support the clinical relevance of ANS function, HRV should primarily be regarded as an indicator of autonomic and emotion-regulatory capacity, rather than a direct treatment target. Its potential prognostic and mechanistic utility lies in informing individualized treatment monitoring and identifying patients who may benefit from interventions that indirectly enhance vagal regulation through psychotherapeutic or behavioral means. Following this thought, restoring integrative functioning of the ANS, may show beneficial in enabling meaningful clinical change in psychiatric symptoms. Respective interventions may comprise regular physical activity and exercise [40], changes in nutrition and diet [41] or directly targeting vagal function by means of vagus nerve stimulation [42]. Additionally, psychotherapeutic and psychiatric interventions also represent clinically relevant strategies to enhance HRV and overall autonomic regulation. Psychotherapeutic interventions targeting emotion regulation may also support autonomic regulatory capacity over time [35]. Integrating such approaches may therefore offer a more comprehensive, multimodal framework for promoting long-term autonomic and emotional stabilization in adolescents with NSSI.

From a translational perspective, the integration of HRV monitoring in adolescents within clinical settings involves both ethical and practical considerations, including informed and voluntary participation of adolescents alongside parental consent as well as logistical constraints of implementing psychophysiological assessments in routine care [43]. These factors may reduce participation rates and limit the feasibility of repeated measurements in routine care contexts. An additional ethical concern is that a primarily biological perspective may contribute to over-medicalization by re-classifying normal developmental variation as clinical pathology, which underscores the importance of interpreting physiological data within psychosocial and developmental context [44]. Several limitations should be considered. Attrition was substantial—especially at follow-up 2—and therefore represents a key limitation of the present study. Analyses were conducted using all available data under the assumption of data missing at random (MAR). Since this assumption cannot be formally tested, baseline demographic and clinical characteristics of participants with follow-up data were compared descriptively to those without follow-up participation, and no systematic differences were observed in age, sex, baseline symptom severity, or autonomic indices. Nonetheless, potential bias due to non-random dropout cannot be fully excluded and should be examined in future studies with larger longitudinal samples.

The lack of a control group limits the ability to distinguish clinical effects from normative developmental changes. Although key baseline confounders were controlled, other relevant factors, including lifestyle influences such as caffeine intake or recent physical activity, were not modeled as time-varying covariates have been omitted to reduce model complexity and avoid overfitting. The use of only three annual measurements constrains the detection of dynamic patterns and short-term or non-linear changes. High within-person variability likely obscured individual slopes, and model limitations—such as linear assumptions, challenges in capturing individual trajectories, added complexity from lagged effects, and potential for better model fit—may have further impacted the accuracy and generalizability of results.

## Conclusion

This study provides evidence that parasympathetic vagal activity, indexed by HRV, is associated with subsequent changes in clinical outcomes in adolescents engaging in NSSI. Increases in HRV were linked to improvements in depression, BPD symptoms, and global functioning, supporting HRV’s role as a biomarker of self-regulation and general psychopathology [41]. These findings align with models emphasizing emotional dysregulation as central to the development of mental health disorders [37], [42]. Notably, changes in HRV were not directly associated with changes in NSSI, suggesting that underlying self-regulatory dysfunction [43] may not manifest directly in behavioral measures. Consistent with prior research, our results highlight HRV’s potential utility in early detection, monitoring treatment progress, and informing personalized interventions [44–47]. However, limitations such as high attrition and the absence of a control group warrant caution and underscore the need for further research to validate and extend these findings.

## Data Availability

Due to the nature of this research project, participants did not provide consent for their data to be shared publicly, so supporting data is not publicly available. However, anonymized data can be made available upon request from the corresponding author.
